# Supplementary material for: Effect of Vitamin D Supplements on Relapse of Digestive Tract Cancer with Tumor Stromal Immune Response: A Secondary Analysis of the AMATERASU Randomized Clinical Trial
Source: Cancers (Basel). 2021 Sep 20;13(18):4708. doi: 10.3390/cancers13184708 (PMC8470811; doi:10.3390/cancers13184708)
Supplement: Supplementary file 1 [file cancers-13-04708-s001.zip › cancers-1368359-supplementary.pdf]

Supplementary Online Content

Effect of Vitamin D Supplements on Relapse of Digestive Tract Cancer with Tumor Stromal Immune Response: A Secondary Analysis of the AMATERASU Randomized Clinical Trial

Authors: Taisuke Akutsu, MD, PhD; Kazuki Kanno, MD; Shinya Okada, MD, PhD; Hironori Ohdaira, MD, PhD; Yutaka Suzuki, MD, PhD; and Mitsuyoshi Urashima, MD, MPH, PhD

Figure S1. Patient flowchart through the present post hoc analysis --- page 2

Figure S2. Image analysis of CD3+, CD4+, CD8+, CD45RO+, and CD56+ cells in digestive tract cancers --- page 3

Table S1. Associations among the densities of CD3+, CD4+, CD8+, CD45RO+, and CD56+ tumor-infiltrating lymphocytes --- page 4

Figure S3. Scatter plots and associations among the densities of CD3+, CD4+, CD8+, CD45RO+, and CD56+ in tumor stroma --- page 5

Table S2. Patients' characteristics in subgroups stratified by infiltration of immune cells in tumor stroma --- page 6 - 10

Table S3. Patients' characteristics stratified by vitamin D vs. placebo --- page 11 - 13

Figure S4. Subdistribution hazard ratios of relapse according to higher and lower halves of each subset for tumor-infiltrating lymphocytes in the vitamin D group compared with the placebo group --- page 14

Figure S5. Hazard ratios of death according to higher and lower halves of each subset for tumor-infiltrating lymphocytes in the vitamin D group compared with the placebo group --- page 15

Figure S6. Hazard ratios of death according to the higher and lower halves of each subset for tumor-infiltrating lymphocytes in the vitamin D group compared with the placebo group --- page 16

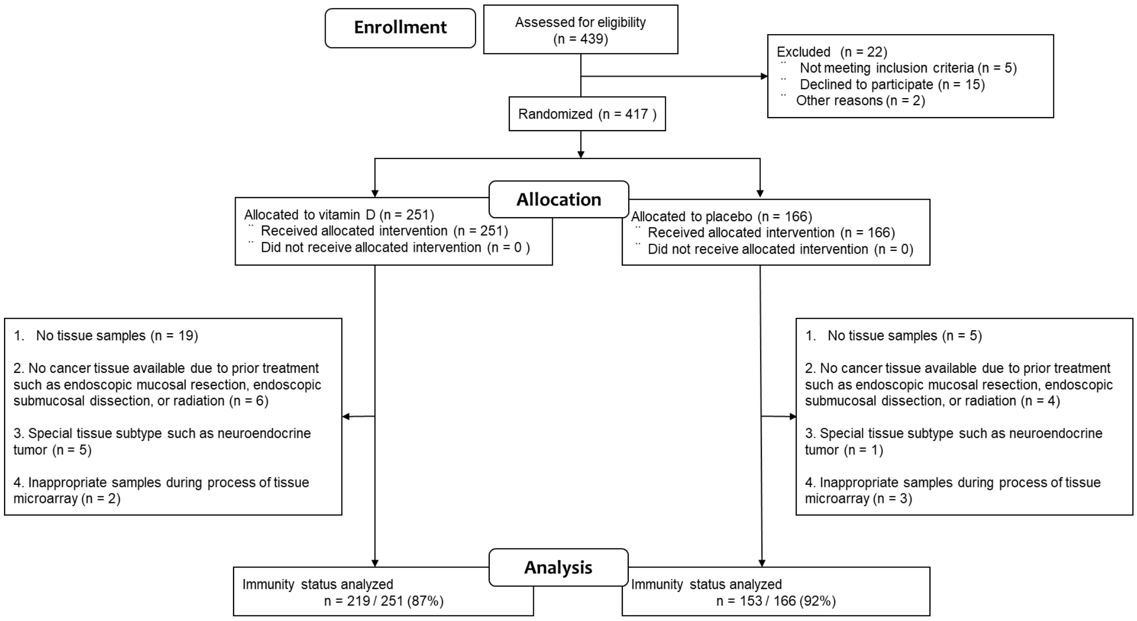

Figure S1. Patient flowchart through the present post hoc analysis

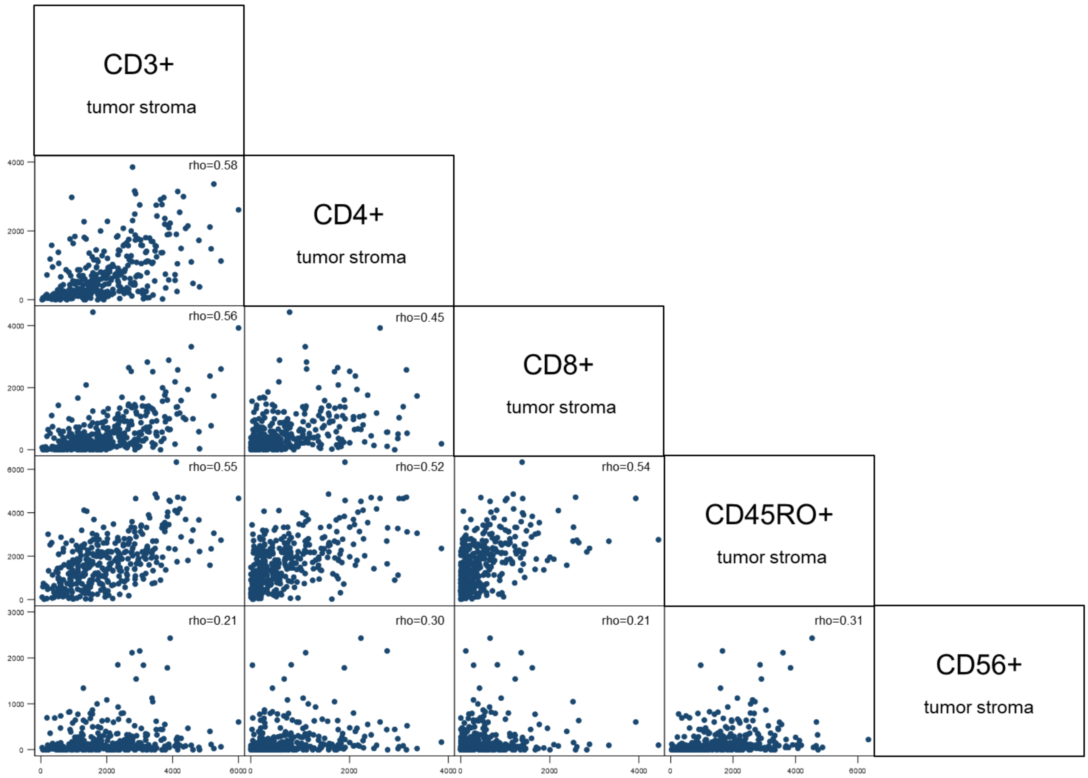

Figure S2. Scatter plots and associations among the densities of CD3+, CD4+, CD8+, CD45RO+, and CD56+ in tumor stroma

Spearman's rank correlation coefficient (rho) is used to quantify the strength of associations of each density of CD3+, CD4+, CD8+, CD45RO+, and CD56+ cells in tumor stroma. CD3+, 4+, 8+, and 45RO+ cells are moderately related to each other, whereas CD56+ cells have a weak association with other subsets.

Table S1. Associations among the densities of CD3+, CD4+, CD8+, CD45RO+, and CD56+ tumor-infiltrating lymphocytes

	CD3+ TIL	CD4+ TIL	CD8+ TIL	CD45RO+TIL	CD56+ TIL
**CD3+ TIL**	-				
**CD4+ TIL**	rho = 0.43	-			
**CD8+ TIL**	rho = 0.60	rho = 0.35	-		
**CD45RO+TIL**	rho = 0.39	rho = 0.21	rho = 0.33	-	
**CD56+ TIL**	rho = 0.13	rho = 0.16	n. s.	n. s.	-

* Spearman's rank correlation coefficient (rho) was used to quantify the strengths of associations between two continuous variables: rho ≥ 0.9, very strong; 0.9 > rho ≥ 0.7, strong; 0.7 > rho ≥ 0.4, moderate; 0.4 > rho ≥ 0.1, weak; and rho < 0.1, negligible. n. s. not significant

The distribution of the density of each lymphocytic infiltrate subset (median [interquartile range] number/mm^2) was as follows: CD3+ 364 [143–727], CD4+ 104 [182-1070], CD8+ 125 [34-339], CD45RO+ 551 [222-1098], and CD56+ 40 [0-91]. There were moderate associations between CD3+ and CD4+ cells and between CD3+ and CD8+ cells. In contrast, other combinations had weak or no significant associations with other subsets.

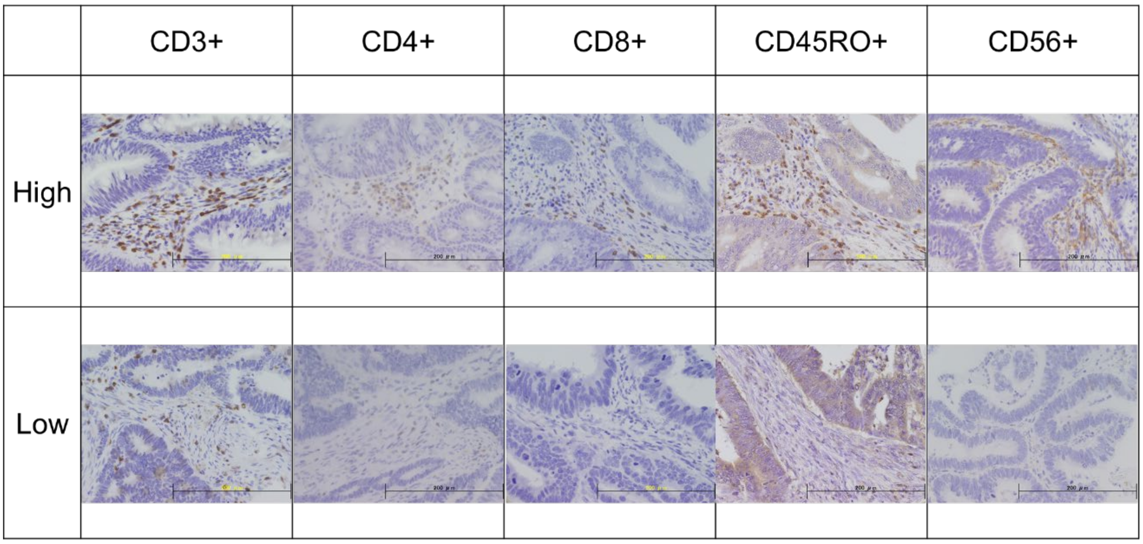

Figure S3. Typical images of CD3+, CD4+, CD8+, CD45RO+, and CD56+ cells

CD3+, CD4, CD8+, CD45RO+, and CD56+ cells in digestive tract cancers. Upper panels show high-density cases representing the higher-half subgroups, and lower panels show the low-cell density cases representing the lower-half subgroups. The distribution of the density of each lymphocytic infiltrate subset (median [interquartile range] number/mm^2) is as follows: CD3+ 1747 [1095-2731], CD4+ 467 [182-1070], CD8+ 305 [93-723], CD45RO+ 1606 [783-2355], and CD56+ 70 [28-208].

Table S2. Patients' characteristics in subgroups stratified by infiltration of immune cells in tumor stroma

	CD3		CD4		CD8		CD45RO		CD56	
	High	Low	High	Low	High	Low	High	Low	High	Low
	N=186	N=186	N=186	N=186	N=186	N=186	N=186	N=186	N=186	N=186
Intervention										
Vitamin D, No. (%)	115 (62)	104 (56)	117 (63)	102 (55)	115 (62)	104 (56)	113 (61)	106 (57)	108 (58)	111 (60)
Placebo, No. (%)	71 (38)	82 (44)	69 (37)	84 (45)	71 (38)	82 (44)	73 (39)	80 (43)	78 (42)	75(40)
25(OH)D [b], No. (%) [a]	N=182	N=184	N=182	N=184	N=181	N=185	N=182	N=184	N=183	N=183
Very low: <10 ng/mL	7 (4)	10 (5)	7 (4)	10 (5)	9 (5)	8 (4)	7 (4)	10 (5)	10 (5)	7 (4)
Low: ≥ 10 and < 20 ng/mL	64 (35)	71 (39)	67 (37)	68 (37)	65 (36)	70 (38)	67 (37)	68 (37)	57 (31)	78 (43)
High: ≥ 20 ng/mL	116 (61)	103 (56)	108 (59)	106 (58)	107 (59)	107 (58)	108 (59)	106 (58)	116 (63)	98 (54)
25(OH)D [b], ng/mL	N=182	N=184	N=182	N=184	N=181	N=185	N=182	N=184	N=183	N=183
median	22	21	21	22	21	21	21	21	22	21
IQR (25%-75%)	(16-27)	(16-27)	(15-27)	(16-27)	(15-27)	(16-27)	(16-27)	(16-27)	(16-28)	(15-26)
Bioavailable 25(OH)D [b], ng/mL, No. (%)	N=155	N=160	N=157	N=158	N=154	N=161	N=157	N=158	N=158	N=157
Low: < median	76 (49)	80 (50)	87 (55)	69 (44)	78 (51)	78 (48)	82 (52)	74 (47)	76 (48)	80 (51)
High: ≥ median	79 (51)	80 (50)	70 (45)	89 (56)	76 (49)	83 (52)	75 (48)	84 (53)	82 (52)	77 (49)
Bioavailable 25(OH)D [b], ng/mL	N=155	N=160	N=157	N=158	N=154	N=161	N=157	N=158	N=158	N=157
median	1.8	1.7	1.6	1.9	1.7	1.7	1.6	1.8	1.8	1.7
IQR (25%-75%)	(1.2-2.6)	(1.3-2.5)	(1.2-2.3)	(1.2-2.8)	(1.2-2.6)	(1.2-2.6)	(1.2-2.4)	(1.3-2.6)	(1.3-2.7)	(1.2-2.5)
Sex, No. (%)										
Male	125 (67)	123 (66)	130 (70)	118 (63)	124 (67)	124 (67)	120 (65)	128 (69)	125 (67)	123 (66)

Female	61 (33)	63 (34)	56 (30)	68 (37)	62 (33)	62 (33)	66 (35)	58 (31)	61 (33)	63 (34)
Age quartile, No. (%) [a]										
Q1, 35-59 y	47 (25)	40 (22)	40 (22)	47 (25)	43 (23)	44 (24)	39 (21)	48 (26)	45 (24)	42 (23)
Q2, 60-65 y	40 (22)	43 (23)	42 (23)	41 (22)	43 (23)	40 (22)	41 (22)	42 (23)	44 (24)	39 (21)
Q3, 66-73 y	46 (25)	56 (30)	56 (30)	46 (25)	51 (27)	51 (27)	55 (30)	47 (25)	51 (27)	51 (27)
Q4, 74-90 y	53 (28)	47 (25)	48 (26)	52 (28)	49 (26)	51 (27)	51 (27)	49 (26)	46 (25)	54 (29)
Body mass index [c] quartile, No. (%) [a]										
Q1, 15.0-19.7 kg/m^2	46 (25)	45 (24)	35 (19)	56 (30)	42 (23)	49 (26)	43 (24)	48 (26)	37 (20)	54 (29)
Q2, 19.8-21.8 kg/m^2	49 (26)	42 (23)	49 (26)	42 (23)	52 (28)	39 (21)	50 (27)	41 (22)	45 (24)	46 (25)
Q3, 21.9-23.7 kg/m^2	42 (23)	53 (29)	46 (25)	49 (26)	45 (24)	50 (27)	47 (25)	48 (26)	48 (26)	47 (26)
Q4, 23.8-37.3 kg/m^2	48 (26)	45 (24)	55 (30)	38 (21)	46 (25)	47 (25)	45 (24)	48 (26)	56 (30)	37 (20)
History of other cancers, No. (%) [a]	6 (3)	7 (4)	6 (3)	7 (4)	6 (3)	7 (4)	7 (4)	6 (3)	8 (4)	5 (3)
Comorbid condition, No. (%) [a]										
Hypertension	62 (33)	80 (43)	66 (35)	76 (41)	66 (35)	76 (41)	71 (38)	71 (38)	73 (39)	69 (37)
Diabetes mellitus	29 (16)	34 (18)	35 (19)	28 (15)	35 (19)	28 (15)	35 (19)	28 (15)	36 (19)	27 (15)
Endocrine disease	23 (12)	26 (14)	25 (13)	24 (13)	24 (13)	25 (13)	22 (12)	27 (15)	29 (16)	20 (11)
Cardiovascular disease	12 (6)	14 (8)	12 (6)	14 (8)	16 (9)	10 (5)	12 (6)	14 (8)	15 (8)	11 (6)
Chronic kidney disease	2 (1)	3 (2)	1 (0.5)	4 (2)	2 (1)	3 (2)	1 (0.5)	4 (2)	0 (0)	5 (3)
Asthma	2 (1)	1 (0.5)	2 (1)	1 (0.5)	1 (0.5)	2 (1)	1 (0.5)	2 (1)	1 (0.5)	2 (1)
Orthopedic disease	0 (0)	2 (1)	0 (0)	2 (1)	1 (0.5)	1 (0.5)	2 (1)	0 (0)	0 (0)	2 (1)
Site of cancer, No. (%) [a]										
Esophagus	13 (7)	21 (11)	12 (6)	22 (12)	12 (6)	22 (12)	14 (8)	20 (11)	5 (3)	29 (16)

Stomach	73 (39)	86 (46)	77 (41)	82 (44)	90 (48)	69 (37)	90 (48)	69 (37)	94 (51)	65 (35)
Small bowel	1 (0.5)	1 (0.5)	2 (1)	0 (0)	2 (1)	0 (0)	2 (1)	0 (0)	2 (1)	0 (0)
Colorectal	99 (53)	78 (42)	95 (51)	82 (44)	82 (44)	95 (51)	80 (43)	97 (52)	85 (46)	92 (49)
Stage, No. (%) [a]										
I	89 (48)	71 (38)	89 (48)	71 (38)	88 (47)	72 (39)	92 (49)	68 (27)	103 (55)	57 (31)
II	46 (25)	55 (30)	46 (25)	55 (30)	46 (25)	55 (30)	51 (27)	50 (27)	44 (24)	57 (31)
III	51 (27)	60 (32)	51 (27)	60 (32)	52 (28)	59 (32)	43 (23)	68 (37)	39 (21)	72 (39)
Pathology, No. (%) [d e]										
Adenocarcinoma										
Well-differentiated	111 (60)	92 (49)	110 (59)	93 (50)	101 (54)	102 (55)	99 (53)	104 (56)	108 (58)	95 (51)
Moderately differentiated	82 (44)	76 (41)	71 (38)	87 (47)	88 (47)	70 (38)	80 (43)	78 (42)	75 (40)	83 (45)
Poorly differentiated	34 (18)	37 (20)	32 (17)	39 (21)	41 (22)	30 (16)	45 (24)	26 (14)	40 (22)	31 (17)
Signet ring cell	10 (5)	31 (17)	14 (8)	27 (15)	20 (11)	21 (11)	24 (13)	17 (9)	29 (16)	12 (6)
Mucinous	12 (6)	14 (8)	13 (7)	13 (7)	15 (8)	11 (6)	14 (8)	12 (6)	12 (6)	14 (8)
Papillary	8 (4)	7 (4)	6 (3)	9 (5)	5 (3)	10 (5)	6 (3)	9 (5)	7 (4)	8(4)
Squamous cell carcinoma	12 (6)	16 (9)	10 (5)	18 (10)	11 (6)	17 (9)	13 (7)	15 (8)	5 (3)	24 (12)
P53 expression, No. (%) [a f]										
None	24 (13)	45 (24)	28 (15)	41 (22)	29 (16)	40 (22)	31 (17)	38 (20)	32 (17)	37 (20)
Faintly expressed: > 0% & < 10%	42 (23)	35 (19)	37 (20)	40 (22)	44 (24)	33 (18)	40 (22)	37 (20)	43 (23)	34 (18)
Strongly expressed: ≥ 10% & < 50%	31 (17)	27 (15)	37 (20)	21 (11)	33 (18)	25 (13)	38 (20)	20 (11)	30 (16)	28 (15)
Overexpressed: ≥ 50% [g]	89 (48)	79 (42)	84 (45)	84 (45)	80 (43)	88 (47)	77 (41)	91 (49)	81 (44)	87 (47)
Vitamin D receptor expression, N										

o. (%) [a g]										
Q1,	**40 (22)** [i]	**55 (30)**	44 (24)	51 (27)	54 (29)	41 (22)	56 (30)	39 (21)	42 (23)	53 (28)
Q2,	**41 (22)**	**54 (29)**	43 (23)	52 (28)	46 (26)	49 (26)	45 (24)	50 (27)	43 (23)	52 (28)
Q3,	**50 (27)**	**42 (23)**	49 (26)	43 (23)	44 (24)	48 (26)	43 (23)	49 (26)	53 (28)	39 (21)
Q4,	**55 (30)**	**35 (19)**	50 (27)	40 (22)	42 (23)	48 (26)	42 (23)	48 (26)	48 (26)	42 (23)
Median	**208** [j]	**159**	195	167	171	189	165	189	195	165
(IQR)	**(115-285)**	**(90-245)**	(110-280)	(90-260)	(90-265)	(111-274)	(90-265)	(110-275)	(110-274)	(90-265)
Ki-67 expression, No. (%) [a h]										
Q1,	**19 (10)** [k]	**45 (24)**	**22 (12)** [l]	**42 (23)**	31 (17)	33 (18)	24 (13)	40 (22)	27 (15)	37 (20)
Q2,	**52 (28)**	**67 (36)**	**62 (33)**	**57 (31)**	57 (31)	62 (33)	62 (33)	57 (31)	58 (31)	61 (33)
Q3,	**32 (17)**	**30 (16)**	**32 (17)**	**30 (16)**	34 (18)	28 (15)	36 (19)	26 (14)	31 (17)	31 (17)
Q4,	**83 (45)**	**44 (24)**	**70 (38)**	**57 (31)**	64 (34)	63 (34)	64 (34)	63 (34)	70 (38)	57 (31)
Median (IQR)	**60 (50-70)** [m]	**50 (40-60)**	**60 (49-70)** [n]	**50 (40-70)**						
Adjuvant chemotherapy, No. (%)[a]	**54 (29)** [o]	**77 (41)**	61 (33)	70 (38)	60 (32)	71 (38)	**52 (28)**[m]	**79 (42)**	57 (31)	74 (40)

[a] Percentages may not sum to 100% because of rounding.

[b] Not measured in some patients.

[c] Not measured in some patients. Calculated as weight in kilograms divided by height in meters squared.

[d] Since many patients had multiple histopathological components, histopathological subgroups were not mutually exclusive of each other.

[e] Histopathological subtypes were not mutually exclusive, since there could be multiple subtypes.

[f.] p53-positive was defined as a positive nuclear percentage in the tumor epithelium greater than 10%.

[g] VDR was defined as a score using a semiquantitative scoring system, and Ki-67 as a positive nuclear staining percentage in the tumor epithelium.

[h] Bioavailable 25(OH)D was calculated using serum concentrations of 25(OH)D, vitamin D binding protein (DBP), albumin, and a combination of DBP single-nucleotide polymorphisms.

[i] Chi-squared test: $P = .026$.

[j] Mann–Whitney test: $P = .0025$.

[k] Chi-squared test: $P < .001$.

[l] Chi-squared test: $P = .049$.

[m] Mann–Whitney test: $P < .0001$.

[n] Mann–Whitney test: $P = .001$.

[o] Chi-squared test: $P = .001$.

Table S3. Patients' characteristics stratified by vitamin D vs. placebo

N=372	Vitamin D<br>N=219	Placebo<br>N=153
25(OH)D, n (%)	N=216	N=150
Low: <20 ng/mL	87 (40)	65 (43)
Middle: ≥ 20 and ≤ 40 ng/mL	125 (58)	84 (56)
High: > 40 ng/mL	4 (2)	1 (0.7)
25(OH)D, ng/mL	N=216	N=150
Median<br>IQR (25%-75%)	22<br>(17-27)	21<br>(14-26)
Bioavailable 25(OH)D, ng/mL	N=187	N=128
Low: < median	84 (45)	72 (56)
High: ≥ median	103 (55)	56 (44)
Bioavailable 25(OH)D, ng/mL	N=187	N=128
Median<br>IQR (25%-75%)	1.9<br>(1.3-2.8)	1.6<br>(1.1-2.3)
Sex, n (%)		
Male	152 (69)	96 (63)
Female	67 (31)	57 (37)
Age quartile, n (%)		
Q1, 35-59 y	43 (20)	44 (29)
Q2, 60-65 y	45 (21)	38 (25)
Q3, 66-73 y	57 (26)	45 (29)
Q4, 74-90 y	74 (34)	26 (17)
Body mass index quartile, n (%)		
Q1, 15.0-19.7 kg/m^2	57 (26)	34 (22)
Q2, 19.8-21.8 kg/m^2	53 (24)	38 (25)
Q3, 21.9-23.7 kg/m^2	53 (24)	42 (27)
Q4, 23.8-37.3 kg/m^2	54 (25)	39 (25)
History of other cancers, n (%)	7 (3)	6 (4)
Comorbid condition, n (%)		
Hypertension	89 (41)	53 (35)
Diabetes mellitus	39 (18)	24 (16)
Endocrine disease	31 (14)	18 (12)

**Cardiovascular disease**	17 (8)	9 (6)
**Chronic kidney disease**	4 (2)	1 (0.7)
**Asthma**	3 (1)	0 (0)
**Orthopedic disease**	1 (0.5)	1 (0.7)
**Site of cancer, n (%)**		
**Esophagus**	20 (9)	14 (9)
**Stomach**	92 (42)	67 (44)
**Small bowel**	1 (0.5)	1 (0.7)
**Colorectal**	106 (48)	71 (46)
**Stage, n (%)**		
**I**	100 (46)	60 (39)
**II**	55 (25)	46 (30)
**III**	64 (29)	47 (31)
**Pathology**		
**Adenocarcinoma, n (%)**		
**Well-differentiated**	126 (58)	77 (50)
**Moderately differentiated**	90 (41)	68 (44)
**Poorly differentiated**	38 (17)	33 (22)
**Signet ring cell**	17 (8)	24 (16)
**Mucinous**	18 (8)	8 (5)
**Papillary**	10 (5)	5 (3)
**Squamous cell carcinoma, n (%)**	17 (8)	11 (7)
**P53 expression, n (%)**		
**None**	36 (16)	33 (22)
**Faintly expressed: > 0% & < 10%**	44 (20)	33 (22)
**Strongly expressed: ≥ 10% & < 50%**	31 (14)	27 (18)
**Overexpressed: ≥ 50%**	108 (49)	60 (39)
**Vitamin D receptor expression, n (%)**		
**Q1,**	60 (27)	35 (23)
**Q2,**	56 (26)	39 (25)
**Q3,**	53 (24)	39 (25)
**Q4,**	50 (23)	40 (26)
**Ki-67 expression, n (%)**		
**Q1,**	38 (17)	26 (17)
**Q2,**	76 (35)	43 (28)

**Q3,**	32 (15)	30 (20)
**Q4,**	73 (33)	54 (35)
**Infiltration of immune cells, n (%)**		
**CD3+**	115 (53)	71(46)
**CD4+**	117 (53)	69 (45)
**CD8+**	115 (53)	71 (46)
**CD45RO+**	113 (52)	73 (48)
**CD56+**	108 (49)	78 (51)
**Adjuvant chemotherapy, n (%)**	74 (34)	57 (37)

Subgroup	SHR	(95%CI)	Vitamin D (%)	Placebo (%)	*P*-interaction
CD3+High TIL	1.01	(0.41 – 2.46)	12 (11)	8 (11)	.55
CD3+Low TIL	0.72	(0.41 - 1.25)	24 (22)	25 (33)	
CD4+High TIL	0.72	(0.32 - 1.61)	13 (12)	11 (16)	.80
CD4+Low TIL	0.82	(0.46 - 1.47)	22 (22)	22 (29)	
CD8+High TIL	0.73	(0.35 - 1.52)	15 (14)	14 (19)	.83
CD8+Low TIL	0.81	(0.44 – 1.50)	21 (19)	19 (25)	
CD45RO+High TIL	0.69	(0.37 - 1.31)	19 (18)	19 (26)	.72
CD45RO+Low TIL	0.84	(0.40 - 1.73)	16 (15)	13 (19)	
CD56+High TIL	0.56	(0.27 - 1.15)	13 (13)	18 (22)	.37
CD56+Low TIL	0.88	(0.46 - 1.71)	21 (19)	115 (21)	

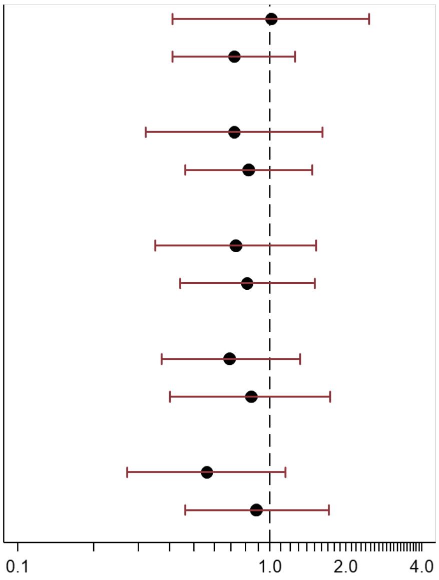

Figure S4. Subdistribution hazard ratios of relapse according to higher and lower halves of each subset for tumor-infiltrating lymphocytes in the vitamin D group compared with the placebo group

To evaluate the effects of vitamin D supplementation on relapse as the primary outcome, cumulative incidence functions were applied by considering patient deaths due to causes other than cancer relapse as a competing risk using subdistribution hazard ratios (SHRs) and 95% confidence intervals (CIs). P for interaction was analyzed by two-way interaction tests comparing the higher-half and lower-half subgroups of each subset of tumor-infiltrating lymphocytes (TILs).

Subgroup	HR	(95%CI)	Vitamin D (%)	Placebo (%)	*P*-interaction
CD3+High	0.54	(0.27 - 1.12)	14 (12)	16 (23)	.18
CD3+Low	1.00	(0.58 - 1.71)	30 (29)	24 (29)	
CD4+High	0.72	(0.37 - 1.40)	19 (16)	16 (23)	.67
CD4+Low	0.85	(0.48 - 1.49)	25 (25)	24 (29)	
CD8+High	0.60	(0.32 - 1.13)	19 (17)	19 (27)	.26
CD8+Low	0.97	(0.54 - 1.73)	25 (24)	21 (26)	
CD45RO+High	0.55	(0.27 - 1.12)	14 (12)	16 (22)	.19
CD45RO+Low	0.98	(0.58 - 1.68)	30 (28)	24 (30)	
CD56+High	0.42	(0.21 - 0.87)	12 (11)	19 (24)	.03
CD56+Low	1.13	(0.65 - 1.97)	32 (29)	21 (28)	

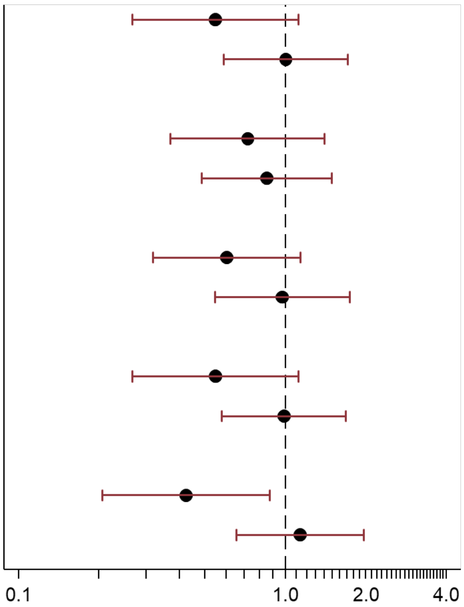

Figure S5. Hazard ratios of relapse or death according to higher and lower halves of each subset for tumor-infiltrating lymphocytes in the vitamin D group compared with the placebo group

To evaluate the effects of vitamin D supplementation on relapse or death, a Cox proportional hazards model was used to determine hazard ratios (HRs) and 95% CIs for relapse or death. P for interaction was analyzed by two-way interaction tests comparing the higher-half and lower-half groups of each subset of tumor-infiltrating lymphocytes (TILs).

Subgroup	HR	(95%CI)	Vitamin D (%)	Placebo (%)	*P*-interaction
CD3+High	0.75	(0.31 - 1.85)	10 (9)	9 (13)	.33
CD3+Low	1.29	(0.65 – 2.56)	23 (22)	13 (16)	
CD4+High	0.80	(0.36 – 1.76)	14 (12)	11 (16)	.31
CD4+Low	1.37	(0.65 – 2.89)	19 (19)	11 (13)	
CD8+High	0.74	(0.35 – 1.58)	15 (13)	12 (17)	.21
CD8+Low	1.49	(0.69 – 3.24)	18 (17)	10 (12)	
CD45RO+High	0.83	(0.36 – 1.89)	13 (12)	10 (14)	.43
CD45RO+Low	1.29	(0.63 – 2.63)	20 (19)	12 (15)	
CD56+High	0.48	(0.19 – 1.20)	8 (7)	11 (14)	.03
CD56+Low	1.69	(0.83 – 3.44)	25 (23)	11 (15)	

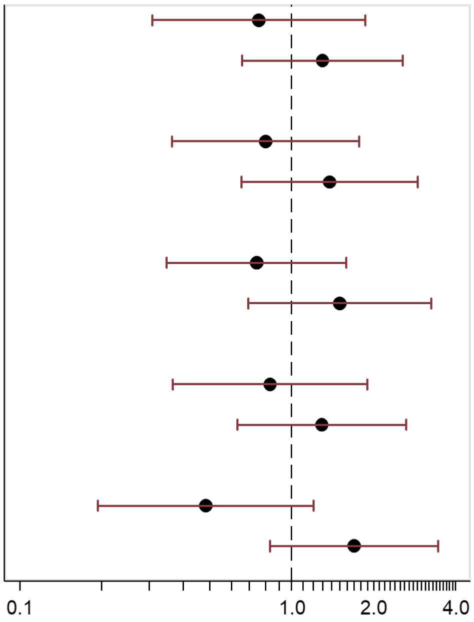

Figure S6. Hazard ratios of death according to the higher and lower halves of each subset for tumor-infiltrating lymphocytes in the vitamin D group compared with the placebo group

To evaluate the effects of vitamin D supplementation on death, a Cox proportional hazards model was used to determine hazard ratios (HRs) and 95% CIs for death. P for interaction was analyzed by two-way interaction tests comparing the higher-half and lower-half subgroups of each subset of tumor-infiltrating lymphocytes (TILs).
